# Endoscopic Appearance of the Gastroesophageal Valve and Competence of the Cardia

**DOI:** 10.1155/DTE.2.147

**Published:** 1996

**Authors:** T. Ismail, J. Bancewicz, J. Barlow

**Affiliations:** University of Manchester Department of Surgery Hope Hospital Salford M6 8HD United Kingdom

## Abstract

The endoscopic appearance of the gastroesophageal valve, viewed by the retroflexed gastroscope, has been studied in 51 patients with and without reflux esophagitis. Esophagitis was graded according to its severity, and the yield pressure (YP) was measured in all patients to assess the competence of the cardia. There was a close relationship between the YP and the grades of the gastroesophageal valve. YP was significantly lower in patients with endoscopic oesophagitis than in patients with no evidence of reflux esophagitis (*p* <0.0001). An increased abnormality of the gastroesophageal valve was associated with all grades of esophagitis and with a low YP. The valve mechanism at the cardia has an important role in determining its competence. YP is possibly a measure of the flap valve component of the gastroesophageal junction.


*Diagnostic and Therapeutic Endoscopy*, 1996, Vol. 2, pp. 147–150
Reprints available directly from the publisher
Photocopying permitted by license only

© 1996 OPA (Overseas Publishers Association)
Amsterdam B.V. Published in The Netherlands
by Harwood Academic Publishers GmbH
Printed in Singapore

### Endoscopic Appearance of the Gastroesophageal Valve and Competence of the Cardia

T. ISMAIL, J. BANCEWICZ and J. BARLOW

*University of Manchester, Department of Surgery, Hope Hospital, Salford, United Kingdom*

*(Received September 30, 1993; in final form January 24, 1994)*

The endoscopic appearance of the gastroesophageal valve, viewed by the retroflexed gastroscope, has been studied in 51 patients with and without reflux esophagitis. Esophagitis was graded according to its severity, and the yield pressure (YP) was measured in all patients to assess the competence of the cardia. There was a close relationship between the YP and the grades of the gastroesophageal valve. YP was significantly lower in patients with endoscopic oesophagitis than in patients with no evidence of reflux esophagitis ($p < 0.0001$). An increased abnormality of the gastroesophageal valve was associated with all grades of esophagitis and with a low YP. The valve mechanism at the cardia has an important role in determining its competence. YP is possibly a measure of the flap valve component of the gastroesophageal junction.

**KEY WORDS:** Gastroesophageal valve, yield pressure, reflux esophagitis

### INTRODUCTION

Since the demonstration of a high-pressure zone in the lower esophagus by Fyke et al.,[1] the lower esophageal sphincter (LOS) pressure has been used to assess the competence of the antireflux mechanism. This has persisted despite a poor correlation between LOS pressure and gastroesophageal reflux (GOR).[2] It seems unlikely that such an important physiological function would be vested solely in such a weak sphincter, and other mechanisms might be expected to contribute to the competence of the cardia. The acute angle between the esophagus and the stomach was described by His,[3] and he suggested that it increased with gastric distention. Marchand[4] confirmed this finding and concluded that the altered angle is a factor of paramount importance in the genesis of GOR. Some investigators have failed to demonstrate a flap valve at the cardia.[5,6] Others stressed the importance of gastric sling fibers in maintaining the gastroesophageal angle and preventing GOR.[7,8]

Intraluminal esophageal pressure is lower than abdominal pressure and, at rest, closure of the abdominal segment is maintained by external compression. The esophagus is compressed from the front against the spine. Computed tomographic examination of the intra-abdominal esophagus appears to confirm this flattened cross-section rather than the circular section, which would be expected of sphincter contraction.[9]

It has been suggested recently that the gastroesophageal valve is a flat musculomucosal structure created by the angle of His.[10] The intact valve in the living human is a complex structure that allows free flow of food and fluid into the stomach but presents a highly effective barrier against retrograde flow. Hill et al. have suggested that the valve and the sphincter can be seen working together—the valve does the heavy work, withstanding the large pressures exerted against the gastroesophageal Junction. The sphincter does the discriminatory work, discriminating between gas, fluid, and solids.

This study was designed to assess the endoscopic appearances of the gastroesophageal valve and to examine the relationship between these appearances and the competence of the cardia. We used yield pressure (YP) measurement as a method for assessment of the competence of the cardia as our previous studies have shown an inverse linear relationship between esophageal acid exposure and yield pressure of the cardia.[11]

Address for correspondence: J. Bancewicz, University of Manchester, Department of Surgery, Hope Hospital, Salford M6 8HD, United Kingdom.

### PATIENTS AND METHODS

The study included 51 patients (27 men and 24 women). The median age was 48 years (range 23 to 78 years). Patients were studied during routine endoscopy examinations. Those with esophageal cancer, gastric cancer, or achalasia were excluded. Those with benign strictures were included if the gastroscope could pass through the stricture and the stricture was above the cardia (e.g., Barrett's esophagus). The YP was measured in all patients. Esophagitis was graded according the AFP classification suggested by Bancewicz et al. and modified by the International Society for Diseases of the Esophagus.[13] P denotes pathology and was graded according to severity as follows: P0, no macroscopic mucosal abnormality; P1, isolated or nonconfluent erosive lesions of the mucosa; P2, circumferential or confluent erosive lesions in the mucosa; P3, chronic lesions involving the wall, e.g., stricture, short esophagus, and penetrating ulcer.

The valvular appearance of the cardia visualized from below by the retroflexed gastroscope was described using a new grading system, V grades (Fig. 1). This classification is based on the actual valve appearance of the cardia closing around the gastroscope and the presence or absence of hiatus hernia as follows: **V0, no hiatus hernia and normal valve appearance; V1, small hiatus hernia, the cardia closed around the gastroscope; V2, open cardia with minimum distention and no hiatus hernia; V3, open cardia and hiatus hernia.**

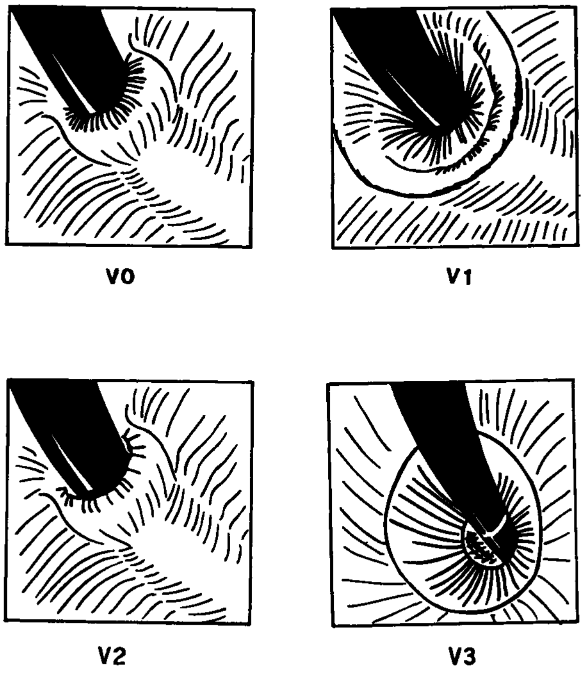

**Figure 1** Artist's impression of the grades of the gastroesophageal valve (V grades) viewed through the retroflexed gastroscope.

A Fujinon UG1 F4 gastroscope with external diameter of 11 mm was used, and one surgeon performed all endoscopic examinations in the study. A Lignocaine throat spray was used, and the patient was sedated with diazepam (10 to 20 mg. i.v.) until drowsy. Hyoscine hydrobromide (Buscopan), 10 mg i.v., was also given.

The endoscope was passed into the stomach under direct vision, insufflating as little air as possible. A polyvinyl catheter 230 cm long, 2 mm external diameter, and 1 mm internal diameter, was passed down the biopsy channel, so that approximately 1 cm protruded beyound the distal end of the gastroscope. It was connected to a pressure transducer (Gould P23 ID, California) and was continuously perfused with water at a rate of 0.6 ml/min using a low compliance pneumohydraulic capillary infusion system (Arndorfer Medical Specialities, Wisconsin). The tip of the endoscope was then retroverted and the instrument withdrawn so that the cardia could be visualised from below. The valve appearance was graded by the endoscopist according to the classification mentioned above. The resting gastric pressure (RGP) was recorded using a Polygraph 7E eight channel recorder (Grass Instruments, Quincy, MA). Air was insufflated until rising intragastric pressure forced the cardia open. The opening pressure (OP) at this point was recorded. The YP of the cardia calculated as YP = OP–RGP. All pressure measurements were made independently by another observer who was not aware of the endoscopic findings.

### RESULTS

### Relationship Between the Gastroesophageal Valve and YP

In 22 patients with a normal looking valve, the median YP was 15 mm Hg. YP was progressively reduced with increasing valve abnormality (Fig. 2). In 13 patients with V1 grade the median YP was 8 mm Hg ($p < 0.0004$). In the group with V2 grade the median YP was 4.5 mm Hg, whereas in 10 patients with grossly abnormal valves of V3 grade, the median YP was 0.5 mm Hg.

### Relationship Between Grades of Esophagitis and YP

There was a significant difference in YP between patients with grade 1 esophagitis and those with no evidence of macroscopic esophagitis, ($p < 0.0001$). In five patients with grade 2 and 3 esophagitis, the median YP was similar to the group with grade 1 esophagitis although the numbers are too small for statistical comparison (Fig. 3).

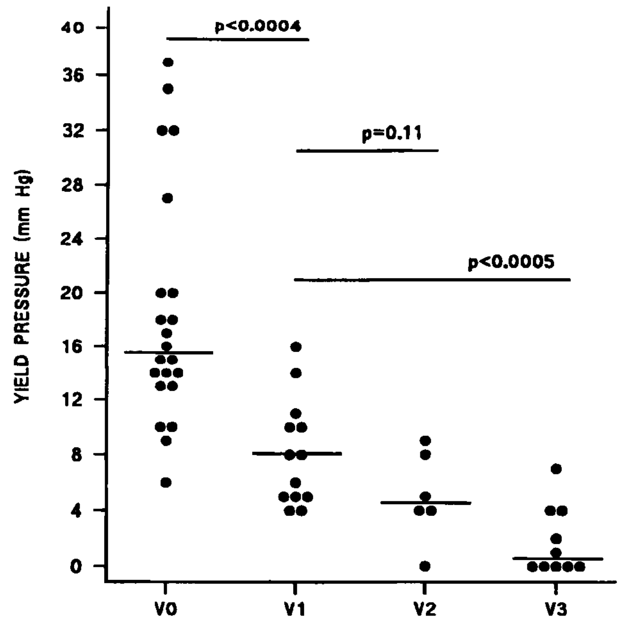

**Figure 2** Relationship between grades of the valve and yield pressure.

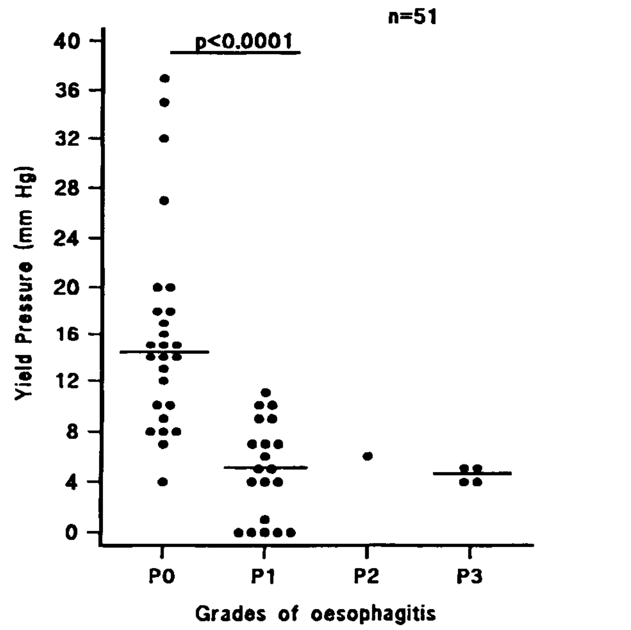

**Figure 3** Relationship between grades of esophagitis and yield pressure.

### Relationship Between the Gastroesophageal Valve and Grades of Esophagitis

The majority of patients with a normal valve had no esophagitis. The different grades of abnormal valves were associated with all grades of esophagitis. Few patients with abnormal valves had no esophagitis and some of these patients were receiving omeprazole treatment at the time of endoscopy (Fig. 4).

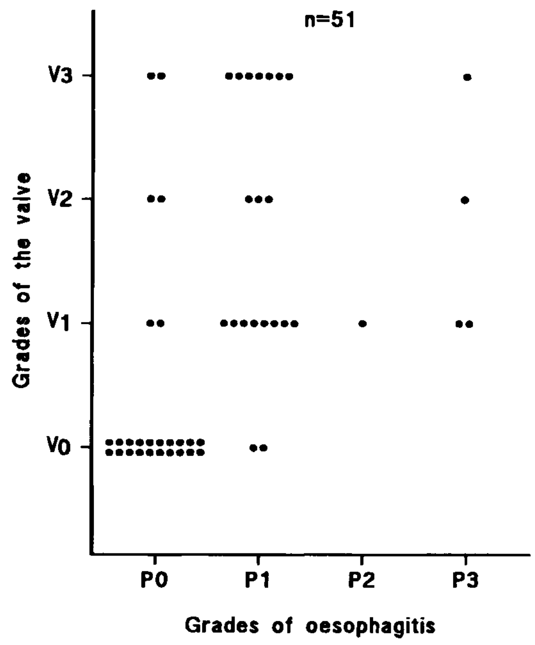

**Figure 4** Relationship between grades of esophagitis and grades of the valve.

### DISCUSSION

In this study we have described a new classification for the appearance of the gastroesophageal valve. We have shown for the first time a significant relationship between the different grades of valve abnormality and the YP measurement. To our knowledge this is the first demonstration of a correlation between the valve mechanism at the gastroesophageal junction and a specific pressure measurement.

Hill and his colleagues[10] have stressed the importance of the gastroesophageal valve and described a different grading system for its appearance; however, these gradings were not correlated with any particular measurement of gastroesophageal competence. This study supports the concept of the flap valve at the gastroesophageal junction as an important factor affecting its competence. We have previously shown that YP is a useful measurement for assessment of the competence of the cardia and that it increases significantly after successful Nissen fundoplication.[11] The results of this study show that abnormal valves are associated with low YP measurement and with all grades of esophagitis.

We believe that the gastroesophageal valve is as important as the LOS pressure in maintaining competence of the cardia. YP is a good measure of the flap valve component of the cardia. Understanding of the factors affecting the competence of the cardia has important implications improved results from antireflux surgery.

### REFERENCES

1. Fyke FE, Code DF, Schlegel JF. The gastro-oesophageal sphincter in healthy human beings. Gastroenterologia (Basel) 1956;86:135–150.
2. Katz PO, Castell DO. Esophageal manometry. In: Castell DO, Wu WC, Ott DJ, eds. Gastroesophageal reflux disease. New York: Futura, 1985:129–138.
3. His W. Studien an geharteten leichen uber form and lagerung des mechlichen magens. Arch Anat Physiol 1903;27:345–367.
4. Marchand P. The gastro-oesophageal sphincter and the mechanism of regurgitation. Br J Surg 1955;42:504–513.
5. Nauta J. The closing mechanism between the oesophagus and the stomach. Gastroenterology 1956;86:219–232.
6. Alder RH, Firme CN, Lanigan JM. A valve mechanism to prevent gastro-oesophageal reflux and oesophagitis. Surgery 1958;44:63–76.
7. Atkinson M, Summerling MD. The competence of the cardia after cardiomyotomy. Gastroenterology 1954;92:23–32.
8. Samelson SL, Weiser HF, Bombeck CT, et al. A new concept in the surgical treatment of gastroesophageal reflux. Ann Surg 1983;197:254–259.
9. Clark J. Hiatus hernia and reflux oesophagitis. In: Hennessy TPJ, Cuschieri A, eds. Surgery of the oesophagus. London: Bailliere Tindall, 1986:173–240.
10. Hill LD, Aye RW, Ramel S. Antireflux surgery: a surgeon's look. Gastroenterol Clin North Am 1990;19:745–775.
11. Ismael T, Bancewicz J. Anatomy of the cardia and gastro-oesophageal reflux. Gut 1992;33 (suppl 2):S62.
12. Bancewicz J, Matthews HR, O'Hanarhan, et al. A comparison of surgically treated reflux patients in two surgical centers. In: Little AG, Ferguson DB, Skinner DB, eds. Diseases of the oesophagus, II. New York: Mount Kiscoe, 1990:177–180.
13. Bancewicz J. What is the place of surgery in the treatment of reflux oesophagitis. Gullet 1993;3 (suppl):85–91.

